# Isonitrile-Derivatized Indole as an Infrared Probe for Hydrogen-Bonding Environments

**DOI:** 10.3390/molecules24071379

**Published:** 2019-04-08

**Authors:** Min You, Liang Zhou, Xinyue Huang, Yang Wang, Wenkai Zhang

**Affiliations:** 1 Department of Physics and Applied Optics Beijing Area Major Laboratory, Center for Advanced Quantum Studies, Beijing Normal University, Beijing 100875, China; youmin@mail.bnu.edu.cn (M.Y.); liangzhou@mail.bnu.edu.cn (L.Z.); xinyuehuang@mail.bnu.edu.cn (X.H.); 2 Department of Chemistry, Beijing Normal University, Beijing 100875, China; wangyang17@mail.bnu.edu.cn

**Keywords:** infrared probe, isonitrile group, indole derivative, hydrogen-bonding environments, infrared spectroscopy

## Abstract

The isonitrile (NC) group has been shown to be a promising infrared probe for studying the structure and dynamics of biomolecules. However, there have been no systematic studies performed on the NC group as an infrared probe, when it is bonded to an indole ring. Here, we systematically study the NC stretching mode of two model compounds, 5-isocyano-1H-indole (5ICI) and 5-isocyano-1-methyl-1H-indole (NM5ICI), using Fourier transform infrared (FTIR) spectroscopy. The NC stretching frequency is shown to be strongly dependent on the polarizability of protic solvents and the density of hydrogen-bond donor groups in the solvent when NC is bonded to an indole ring. Infrared pump–probe studies of 5ICI in DMSO and in EtOH further support that the NC stretching mode could be used as a site-specific infrared probe for local environments when NC is bonded to an indole ring.

## 1. Introduction

Infrared (IR) probes have been widely employed as vibrational reporters to investigate site-specific information for protein conformations, local environments, and dynamics in various biomolecular systems using linear and non-linear spectroscopy [1,2,3,4,5,6,7,8]. Although many probes have been used to provide useful information about proteins or polypeptides, their application in time-resolved non-linear IR spectroscopy has been limited. For example, the stretching mode of nitrile is known to be particularly sensitive to hydrogen-bonding (*H*-bonding) interactions owing to its distinguishable blue-shift in various solvents, but its transition dipole strength is small, especially when it is bonded to an aliphatic carbon, which has made it difficult to obtain a non-linear IR signal with a sufficient signal-to-noise ratio [9,10,11]. The azido group has become a desirable IR probe in biological environments at very low concentration owing to its larger transition dipole strength [12,13,14]. Unfortunately, the application of the azido probe has also been limited owing to its broad bandwidth, short vibrational lifetime, and accidental Fermi resonance [13,14,15]. Recently, the cyanamide group has been reported as an optimized version of the nitrile group, owing to its larger transition dipole strength and longer vibrational lifetime [16]. We have also investigated the diazo group as a site-specific IR probe to detect local hydration environments [17]. Although site-specific IR probes have been widely used in protein structure studies, researchers are still actively working on the development of new IR probes.

Tryptophan (Trp) is the most popular site-specific fluorescent probe that is frequently used at or near sites that are responsible for various protein functions [18,19,20,21]. However, the intrinsic IR-active vibrational mode of Trp cannot be used as a site-specific IR probe. Until recently, cyanotryptophans and *n*-cyanoindoles (*n*-CNI, where *n* = 2–7) have been used as site-specific fluorescent [22,23,24,25] and IR probes [26,27,28], and have provided useful local environmental information. Zhang et al. observed a linear dependence between the nitrile stretching frequency of 5-CNI and the Kamlet–Taft parameters, and demonstrated that 5-CNI could be used as a site-specific IR probe [26]. Rodgers et al. demonstrated that the Fermi resonance of 4-CNI could be used to determine the H-bonding status in a local hydration environment. These developments have made *n*-CNI a site-specific fluorescence and IR probe which can be used to study the local environment [27]. For example, Markiewicz et al. investigated the hydration status around the Trp gate of the influenza A M2 proton channel using a 5-CN–Trp probe [28]. Unfortunately, it is difficult to use the *n*-CNI probe in non-linear IR spectroscopy measurements since the transition dipole strength of the nitrile stretching mode is small. Additionally, the low concentration in a biological sample makes the measurement even harder. Tremendous effort has been dedicated to the development of unnatural amino acid IR probes with greater transition dipole strength [16,17].

**Figure 1:**
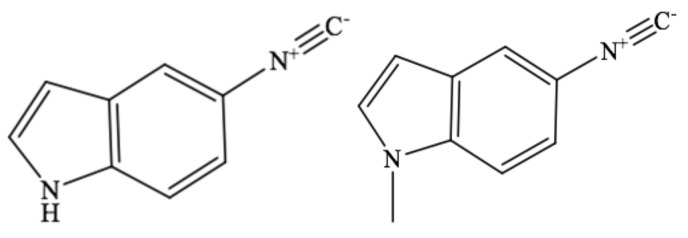
Structures of (**a**) 5-isocyano-1H-indole (5ICI) and (**b**) 5-isocyano-1-methyl-1H-indole (NM5ICI).

Recently, Maj et al. studied two isonitrile group (NC)-derivatized compounds, β-isocyanoalanine (AlaNC) and *p*-isocyanophenylalanine (PheNC), using Fourier transform infrared (FTIR) spectroscopy and femtosecond IR pump–probe spectroscopies [29,30]. They found that the isonitrile stretching frequency is sensitive to H-bonding interactions. Moreover, the transition dipole strength of the NC group was much larger than that of the nitrile group and similar to that of the azido group, and the vibrational lifetime of the NC stretching mode was much longer than those of the nitrile and azido stretching modes [29,30]. This prompted us to study NC-derivatized indoles which could be used as site-specific IR probes for protein structural studies using linear and non-linear IR spectroscopy measurements. To the best of our knowledge, no studies have specifically focused on the quantitative analysis of IR spectroscopy of NC-derivatized indoles. In this study, we systematically studied the solvatochromism of the NC stretching mode of an NC-derivatized indole in different solvents. We first performed FTIR spectroscopy of the two model compounds, 5-isocyano-1H-indole (5ICI, Figure 1a) and 5-isocyano-1-methyl-1H-indole (NM5ICI, Figure 1b) in different solvents. The solvent dependence of the NC stretching frequency and bandwidth were investigated using the Kamlet–Taft empirical parameters (Table 1). The Kamlet–Taft solvent parameters were used to separately measure the solvent polarizability (π*), the hydrogen-bond acceptor (HBA) ability (β), and the hydrogen-bond donor (HBD) ability (α) [31,32]. Furthermore, we studied the relaxation dynamics of 5ICI in DMSO using femtosecond IR pump–probe measurements.

## 2. Results and Discussion

### 2.1. FTIR Spectroscopy

**Figure 2:**
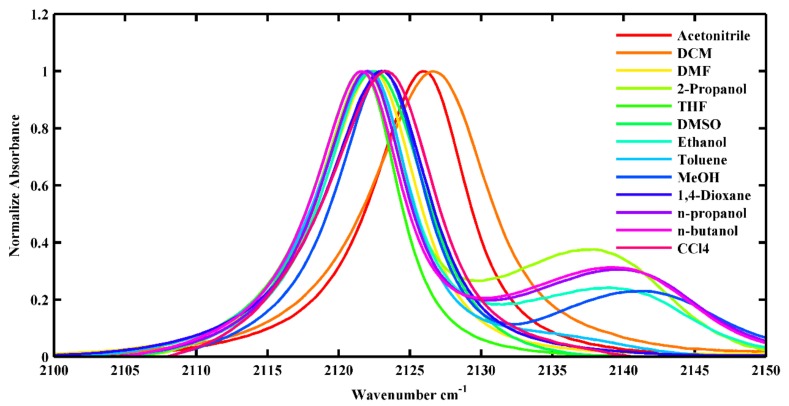
The NC stretching vibration of 5ICI in the studied solvents.

The NC stretching vibration of 5ICI was studied at room temperature in representative pure solvents (Figure 2). The NC stretching frequency of 5ICI was strongly dependent on the solvent. To examine the relationships between the spectral characteristics of NC vibration and the solvent parameters, the lower frequency peak (ω_0_) and relatively weaker blue-shifted peak (ω_1_) of the NC stretching mode in various solvents are listed in Table 1. 

There was one NC stretching peak when 5ICI was dissolved in aprotic solvents, such as THF, toluene, DMSO, DMF, CCl_4_, and 1,4-dioxane. However, there were two stretching peaks when 5ICI was dissolved in the alcohols. Qualitatively, the lower frequency peak and relatively weaker blue-shifted peak in the alcohols could be attributed to the non-H-bonded and H-bonded NC groups [30]. We will discuss the NC stretching mode of the non-H-bonded NC group in the following. The NC stretching frequency of non-H-bonded 5ICI was shifted by approximately 7 cm^−1^ from *n*-octanol to DCM (Table 1). The polarizability of a solvent can induce a local electric field in the NC stretching mode, and this will shift the stretching frequency. There was a shift in the NC stretching frequency of approximately 5 cm^−1^ when the solvent was changed from toluene to DCM, which could result in a decrease in π*, and there was almost no change for the other parameters. Therefore, we could attribute this shift to the change in π*. The HBD ability of the solvent was also shown to play an important role in the NC stretching frequency. For example, a shift of approximately 2 cm^−1^ for the NC stretching frequency was observed when the solvent was changed from THF to MeOH. These two solvents exhibited similar π* and β values, but their α values were quite different. These results suggest that the NC stretching frequency is sensitive to the HBD ability of the solvents.

**Figure 3:**
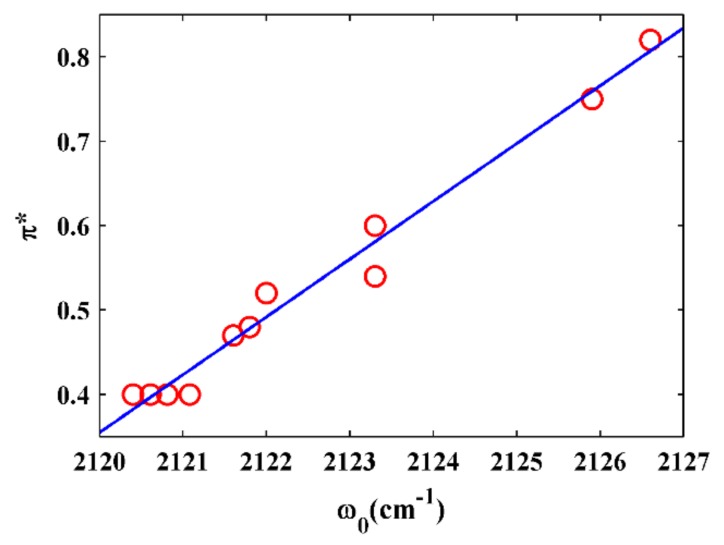
The NC stretching frequencies of 5ICI versus solvent parameter π*. Only frequencies obtained in protic solvents (α ≠ 0) were used.

This simple comparison showed that the NC stretching frequency is sensitive to the polarizability and H-bonding ability of the solvents. A more quantitative evaluation of the FTIR spectra was, therefore, needed to examine the precise contributions of the interactions between the NC group and various solvents. Since there was a significant difference for the NC stretching mode in the protic and aprotic solvents, we will discuss them separately. In the protic solvents, the NC stretching frequencies were in the range from 2120.4 cm^−1^ in *n*-octanol to 2126.6 cm^−1^ in DCM, and the full-width at half maximum (FWHM) of the NC stretching mode were all a little broader than those obtained in the aprotic solvents (Figure 2). Such broadening might be associated with the H-bonding interaction between samples and solvents, which was similar to the results obtained by Maj et al. [29]. The relationships between the NC stretching frequency and the Kamlet–Taft parameter π* for the protic solvents are plotted in Figure 3, and a strong linear correlation was observed. This result suggested that the NC stretching frequency can be a sensitive probe of the polarizability of the protic solvents when the NC group is bonded to an indole ring. However, no such linear correlation was observed when the NC group is bonded to an aliphatic carbon [29]. There were also no good linear relationships between the NC stretching frequency and other Kamlet–Taft parameters (α and β) of the protic solvents (Figure S1). In the aprotic solvents, the NC stretching frequencies were in the range from 2121.6 cm^−1^ in THF to 2123.3 cm^−1^ in CCl_4_, and the FWHM of the NC stretching mode were all approximately 8 cm^−1^. Although the corresponding polarizability of the solvents were significantly different from one another, the central frequency and the bandwidth of the NC stretching mode were very similar. There were no linear relationships between the NC stretching frequency and Kamlet–Taft parameters (π* and β) of the aprotic solvents (Figure S2). These results were very similar to the results when the NC group was bonded to an aliphatic carbon [29]. These results suggested that the NC stretching mode could be a sensitive IR probe in protic environments.

**Figure 4:**
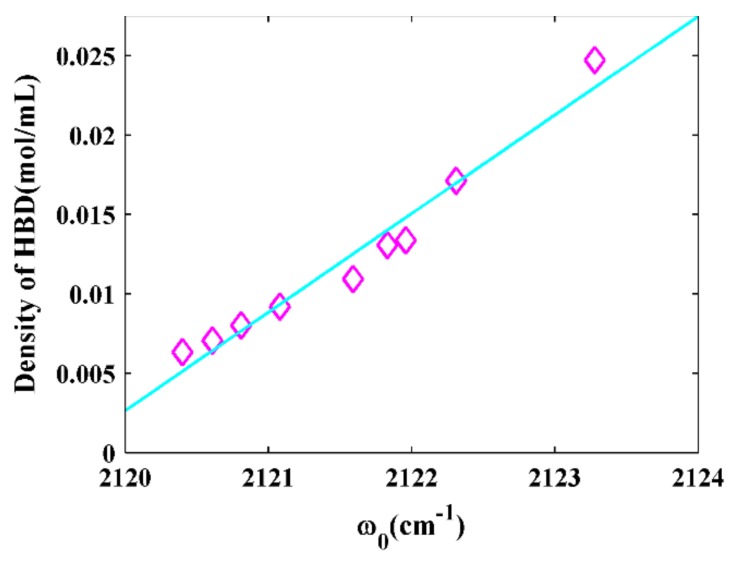
The NC stretching frequencies of 5ICI versus the density of hydrogen-bond donor groups in solvents.

We further investigated the correlation between the NC stretching frequencies in all the studied solvents. There were no linear relationships between the NC stretching frequency and polarizability and the H-bonding ability of the solvents, in accordance with the Kamlet–Taft parameters (Figure S3). However, we found that the NC stretching frequency was linearly dependent (R^2^ = 0.95) on the density of HBD groups in the solvent (Figure 4). The density of HBD groups could be calculated using ρn/M, where ρ is the density of the solvent, M is the molar mass of each solvent, and *n* is the number of HBD groups in every solvent molecule [17,33]. This result was similar to that for the diazo probes and azido probes [17,33]. This suggests that the NC stretching frequency is also sensitive to the local density of HBD groups, especially when there are many local water molecules in the hydrophobic core of a biomolecule. 

**Figure 5:**
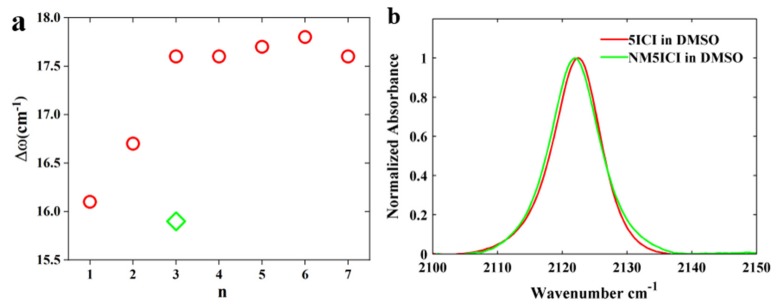
(**a**) The peak separation between the NC stretching frequencies of non-H-bonded and H-bonded NC groups in alcohol solutions versus the carbon chain length (*n*) of the alcohols. (**b**) Comparison of the NC stretching mode of 5ICI and NM5ICI in DMSO.

We will discuss the peak separation when 5ICI was dissolved in the alcohols. The separation between two peaks when the NC was bonded to the indole ring was between 16 and 19 cm^−1^. The separation between two NC peaks was linearly increased with the carbon chain length (*n*) of the alcohols for methanol, ethanol, and 1-propanol (Figure 5a). This may suggest that a longer carbon chain length may stabilize the H-bonding interaction. The peak separation is similar for other alcohols when *n* is larger than 3, this may suggest that more structural conformation may destabilize the H-bonding interaction when *n* > 3. This might also explain why the peak separation in 2-propanol (green diamond in Figure 5a) is smaller than that in the other alcohols. It is worth noting that the peak separation when the NC group was bonded to the indole ring was smaller than the peak separation when the NC group was bonded to an aliphatic carbon, which was around 23 cm^−1^ [29]. This observation suggested that the H-bonding interaction between the NC group and HBD is reduced when the NC group is bonded to the indole ring. Gai and co-workers found that H-bond formations involving the indole N–H group of 5-cyanoindole could be sensed by the nitrile stretching mode and the *H*-bonding occurring at the nitrile and N–H groups had different effects on the nitrile stretching frequency [26]. To test if there was a similar effect for the NC stretching mode of 5ICI, we also carried out static IR measurements on NM5ICI in DMSO because NM5ICI is incapable of forming *H*-bonds with DMSO owing to the added methyl group. As shown (Figure 5b), the NC stretching frequency of NM5ICI in DMSO was centered at 2122.0 cm^−1^, which was red-shifted from that of 5ICI. This finding can at least partially explain why the NC stretching peak separation for the NC group bonded to an indole ring was smaller than that bonded to an aliphatic carbon. We also found that the NC stretching frequency of NM5ICI was linearly dependent (R^2^ = 0.99) on the density of HBD groups in the solvent (Figure S4).

**Figure 6:**
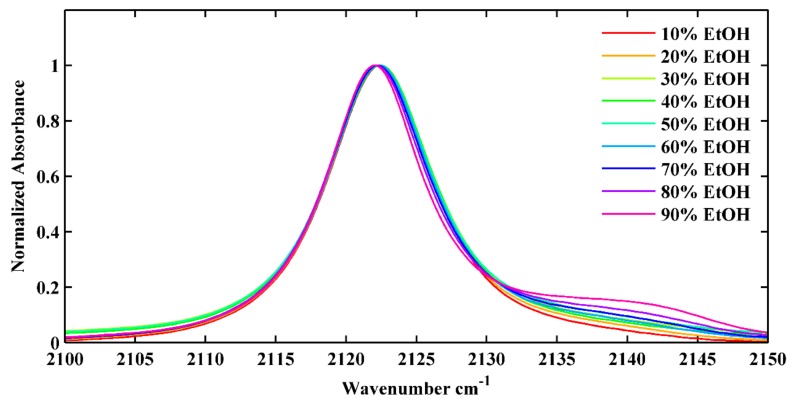
The NC stretching vibration of NM5ICI in DMSO and EtOH mixture with different volume ratios, as indicated.

Finally, we will discuss the relatively weaker blue-shifted peak in the alcohols which were qualitatively attributed to the NC stretching mode of the H-bonded NC group. To further validate this argument, we performed static IR spectroscopy of NM5ICI in binary solvents with various ratios of DMSO and EtOH. We chose NM5ICI instead of 5ICI to eliminate the possible complexity induced by the H-bonding between the solvents and pyrrole N–H group in the indole ring. We chose DMSO because it is a strong HBA but not an HBD solvent. We chose EtOH since it is both a strong HBD and HBA and the solute can hardly dissolve in water. As shown in Figure 6, the lower-frequency NC stretching mode shifted from 2121.9 cm^−1^ to 2121.5 cm^−1^, and the intensity of the relatively weaker blue-shifted peak increased when the ratio of EtOH was increased from 10% to 90%. This result confirmed that the relatively weaker blue-shifted peak in the alcohols could be attributed to the NC stretching mode of the H-bonded NC group. We also investigated the dependence between the NC stretching frequency of the H-bonded NC group and their associated solvent Kamlet–Taft parameters, but no linear correlation was found (Figure S5). There was also no linear correlation between the NC stretching frequency of the H-bonded NC group and the density of the HBD for the NC group (Figure S6).

### 2.2. Polarization-Controlled IR Pump–Probe Spectroscopy

**Figure 7:**
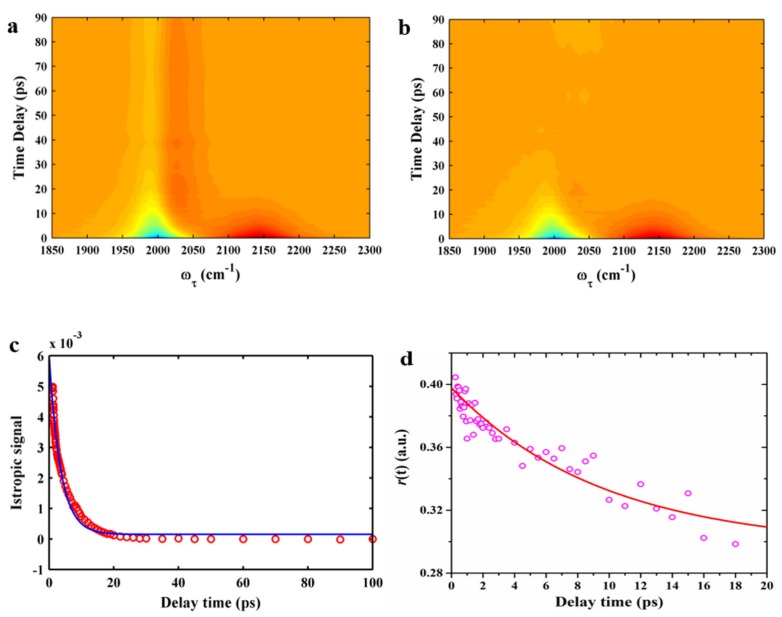
Time- and frequency-resolved isotropic IR pump–probe signals for 5ICI in DMSO before (**a**) and after (**b**) subtraction of the heat contribution. (**c**) Population decay of 5ICI in DMSO. The signal can be fitted to a single exponential function (blue line). (**d**) Anisotropy decays of 5ICI in DMSO. The signal can be fitted to a single exponential function (red line).

We performed polarization-controlled IR pump–probe measurements of the NC stretching mode using 5ICI in DMSO. The frequency-resolved IR pump–probe signals of 5ICI in DMSO are presented in Figure 7a. The IR pump–probe spectra of the NC stretching mode of 5ICI in DMSO clearly revealed the existence of two distinguishable spectral features. A negative band centered at 1996 cm^−1^ could be attributed to ground state bleach (GSB) and stimulated emission (SE). A positive band centered at 2123 cm^−1^ could be attributed to excited-state absorption (ESA). The frequency difference between the GSB and ESA bands was related to vibrational anharmonicity and line broadening. The energy relaxation process should make the signal eventually decay to zero at a longer time delay; however, a finite residual signal was observed, which is similar to that reported in the literature [30]. Maj et al. assigned this residual signal to the pump-induced heating contribution and assumed it followed an exponential rise and subtracted the heating signal from the raw signal [30]. We adopted a similar procedure to remove the heating contribution. The IR pump–probe signals of the NC stretching mode of 5ICI in DMSO after subtraction is presented in Figure 7b, which showed that the heating contribution was removed. The vibrational lifetime of the NC stretching mode of 5ICI in DMSO was obtained from the time profiles of the integrated peak areas of the positive peaks (Figure 7c). The vibrational population decays were fitted by a single exponential function and the resulting time constant was 3.57 ± 0.17 ps. The vibrational lifetime of the NC stretching mode when NC was bonded to indole was several times longer than the azido stretching mode and also longer than that of the CN stretching mode, which was similar to that of NC-derivatized alanine [30]. The orientational relaxation time constant was extracted from the average value of the anisotropic signals in the probe frequency range from 2116 cm^−1^ to 2130 cm^−1^ (Figure 7d). The anisotropy decay was fitted to a single exponential function with a time constant of 14.2 ± 1.4 ps, which was longer than the anisotropy decay time constant of the NC stretching mode for NC-derivatized alanine in DMF [30]. The reason for this may be that the 5ICI molecule is larger than NC-derivatized alanine [30]. Moreover, such a sensitive dependence usually reflects the local solvation structure around the vibrational probe, which suggests that the longer orientational relaxation time of the NC stretching mode of 5ICI could be employed to understand the local intermolecular interactions. All the results indicate that the NC group could be used as a sensitive site-specific IR probe of the local environments.

**Figure 8:**
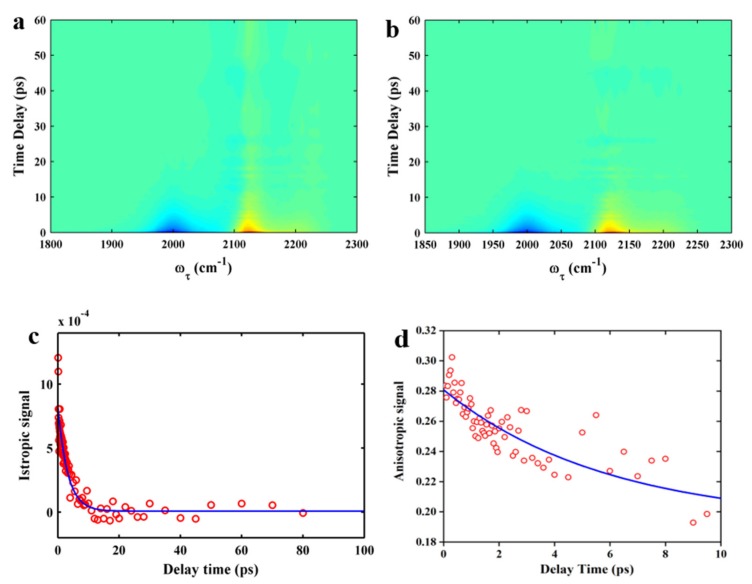
Time- and frequency-resolved isotropic IR pump–probe signals for 5ICI in EtOH before (**a**) and after (**b**) subtraction of the heat contribution. (**c**) Population decay of 5ICI in EtOH. The signal can be fitted to a single exponential function (blue line). (**d**) Anisotropy decays of 5ICI in EtOH. The signal can be fitted to a single exponential function (blue line).

We also performed polarization-controlled IR pump–probe measurements of the NC stretching mode using 5ICI in EtOH as shown in Figure 8a. We also removed the heating contribution as shown in Figure 8b. The vibrational lifetime of the NC stretching mode of 5ICI in EtOH was obtained from the time profiles of the integrated peak areas of the positive peaks (Figure 8c). The vibrational population decays could be fitted by a single exponential function and the resulting time constant was determined to be τ_1_ = 3.21 ± 0.13 ps, which is shorter than that in DMSO. The slowing down of the vibrational relaxation of NC stretching mode could be explained by the hydrogen-bonding (HB) interaction between the 5ICI and solvent, which leads to a decrease of the interaction between the NC stretching vibration and the HB mode. The orientational relaxation time constant was extracted from the average value of the anisotropic signals in the probe frequency range from 2116 cm^−1^ to 2130 cm^−1^ (Figure 8d). The anisotropy decay was fitted to a single exponential function with a time constant of 5.89 ± 0.34 ps, which was much shorter than that of the NC stretching mode for 5ICI in DMSO. All the results indicate that the NC group could be used as a sensitive site-specific IR probe of the local environments.

## 3. Materials and Methods

### 3.1. Materials and Sample Preparation

5-isocyano-1H-indole (5ICI) was purchased from Chemduro Pharm Tech (Wuhan, China) and NM5ICI was purchased from Sigma–Aldrich (St. Louis, MO, USA). The following solvents of HPLC or higher quality were purchased from either Sigma–Aldrich or J&K Scientific (Shanghai, China), and used without further purification: methanol (MeOH), ethanol (EtOH), *n*-octanol, *n*-butanol, 1-propanol, formamide, acetonitrile, dichloromethane (DCM), dimethyl sulfoxide (DMSO), dimethylformamide (DMF), 1,4-dioxane, tetrahydrofuran (THF), toluene, tetrachloromethane (CCl_4_), and hexane. Samples were freshly prepared before use by dissolving either 5ICI or NM5ICI in the desired solvent. The final concentration of the solution was approximately 50 mM for the FTIR and pump–probe measurements, and the sample solution was placed between two CaF_2_ windows separated by a 100-μm or 200-μm spacer.

### 3.2. Spectroscopic Measurements

All FTIR spectra were recorded at room temperature on a Bruker VERTEX 70 spectrometer (Bruker, Karlsruhe, Germany) with a frequency resolution of 0.5 cm^−1^. A solvent background was subtracted from each measured spectrum. Time-resolved spectra were obtained on a polarization-controlled femtosecond IR pump−probe setup. The 800-nm output of a Ti:sapphire regenerative amplifier laser system (Coherent, Santa Clara, CA, USA) was used to pump an optical parametric amplifier (Light Conversion, Lithuania). The generated mid-IR pulse centered at approximately 2100 cm^−1^ was split into pump and probe beams and focused onto the sample. The probe beam after the sample was dispersed by a monochromator onto a mercury cadmium telluride array detector. The pump–probe signal with different probe polarizations relative to the pump beam, parallel S_∥_(t) or perpendicular S_⊥_(t), were selectively measured. The vibrational population and orientational relaxations can be obtained from isotropic and anisotropic signals, respectively: S_iso_(t) = S_∥_(t) + 2S_⊥_(t), and r(t) = [S_∥_(t) − S_⊥_(t)]/S*iso*(t).

## 4. Conclusions

In summary, we systematically studied the IR spectra of the NC model compounds, 5ICI and NM5ICI, in different solvents. We found that the NC stretching frequency was not dependent on the polarizability of aprotic solvents but showed a strong linear correlation with the polarizability of protic solvents. The relatively weaker blue-shifted peak of an NC group in alcohols could be assigned to the NC stretching mode of the H-bonded NC group using binary solvent experiments. Moreover, the NC stretching frequency was found to be linearly correlated with the density of HBD groups in the solvents. We also studied the vibrational population and orientational relaxation time constants of the NC vibration mode for 5ICI in DMSO and EtOH. We found that the NC stretching vibration lifetime in DMSO is similar to that of NC-derivatized alanine in DMF, while it in EtOH is much short than in DMSO. We believe that the NC stretching mode could be employed as a useful IR probe to provide insights about the structure, dynamics, and function of proteins by various IR spectroscopy methods. Especially, it could be used as a site-specific IR probe of local hydration environments.

## Figures and Tables

**Table 1:** The lower frequency peak (ω_0_) and relatively weaker blue-shifted peak (ω_1_) of the isonitrile group (NC) stretching mode in various solvents. Also listed for each solvent are its Kamlet–Taft parameters, π* (polarizability), β (hydrogen-bond acceptor), as well as α (hydrogen-bond donor).

Solvent	ω_0_ cm^−1^	ω_1_ cm^−1^	π*	β	α
*n*-octanol	2120.4	2139.3	0.40	0.81	0.77
THF	2121.6	-	0.58	0.55	0
*n*-butanol	2121.6	2139.2	0.47	0.88	0.79
Toluene	2122.1	-	0.54	0.11	0
2-propanol	2121.8	2137.7	0.48	0.95	0.76
*n*-propanol	2122.0	2139.6	0.52	0.90	0.84
Ethanol	2122.3	2139.0	0.54	0.77	0.83
DMSO	2122.6	-	1	0.76	0
DMF	2122.3	-	0.88	0.69	0
1,4-Dioxane	2123.0	-	0.55	0.37	0
MeOH	2123.3	2139.4	0.6	0.62	0.93
DCM	2126.6	-	0.82	0.1	0.13
CCl_4_	2123.3		0.28	0	0
acetonitrile	2125.9	-	0.75	0.31	0.19
*n*-pentanol	2121.1	2138.8	0.4	0.86	0.84
*n*-hexanol	2120.8	2138.6	0.4	0.94	0.67
*n*-heptanol	2120.6	2138.2	0.39	0.96	0.64

## Supplementary Materials

The following are available online. Figure S1: The NC stretching frequencies of 5ICI versus solvent parameter α or β. Only frequencies obtained in protic solvents (α ≠ 0) were used, Figure S2: The NC stretching frequencies of 5ICI versus solvent parameter π* or β. Only frequencies obtained in aprotic solvents (α = 0) were used, Figure S3: NC stretching frequencies of 5ICI in various solvents versus Kamlet–Taft empirical parameter π*, β, or α, Figure S4: The NC stretching frequencies of NM5ICI versus the density of hydrogen-bond donor groups in solvents (R^2^ = 0.99), Figure S5: The NC stretching frequencies of H-bonded NC group versus Kamlet-Taft empirical parameter π*, β, or α. Only frequencies obtained in protic solvents (α ≠ 0) were used, Figure S6: The NC stretching frequencies of H-bonded NC group versus the density of hydrogen-bond donors of solvents, Figure S7: The NC stretching vibration of NM5ICI in selected solvents.
