# Supplementary material for: Isonitrile-Derivatized Indole as an Infrared Probe for Hydrogen-Bonding Environments
Source: Molecules. 2019 Apr 8;24(7):1379. doi: 10.3390/molecules24071379 (PMC6480494; doi:10.3390/molecules24071379)

**Supplementary Material for:**

# Isonitrile Derivatized Indole as an Infrared Probe for Hydrogen-bonding Environments

**Figure S1** The NC stretching frequencies of 5ICI *versus* solvent parameter α or β. Only frequencies obtained in protic solvents (α ≠ 0) were used.

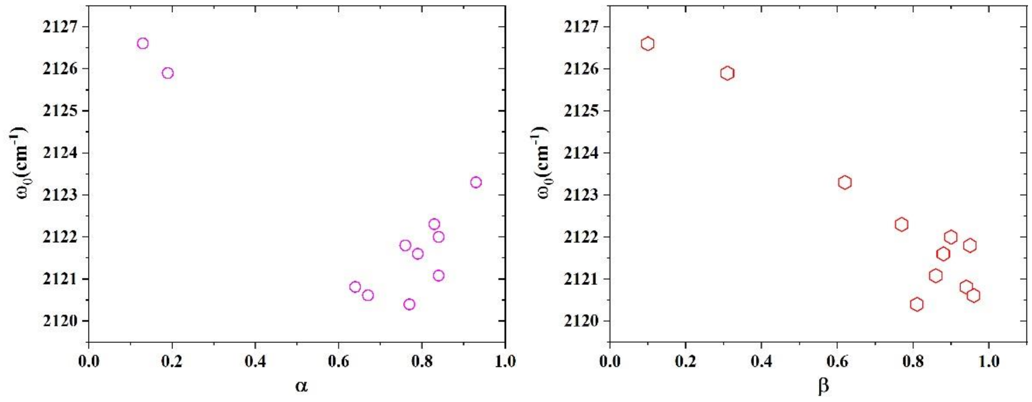


**Figure S2** The NC stretching frequencies of 5ICI *versus* solvent parameter π* or β. Only frequencies obtained in aprotic solvents (α = 0) were used.

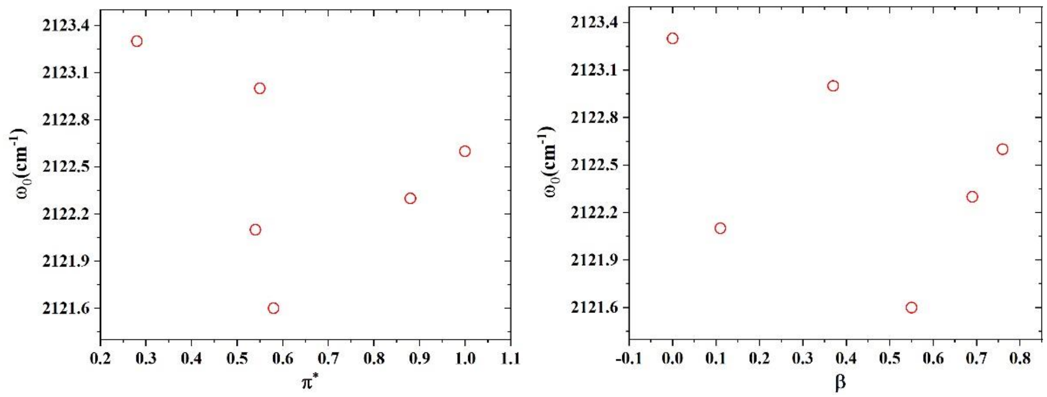

**Figure S3** The NC stretching frequencies of 5ICI in various solvents *versus* Kamlet-Taft empirical parameter $\pi^*$, $\beta$, or $\alpha$.

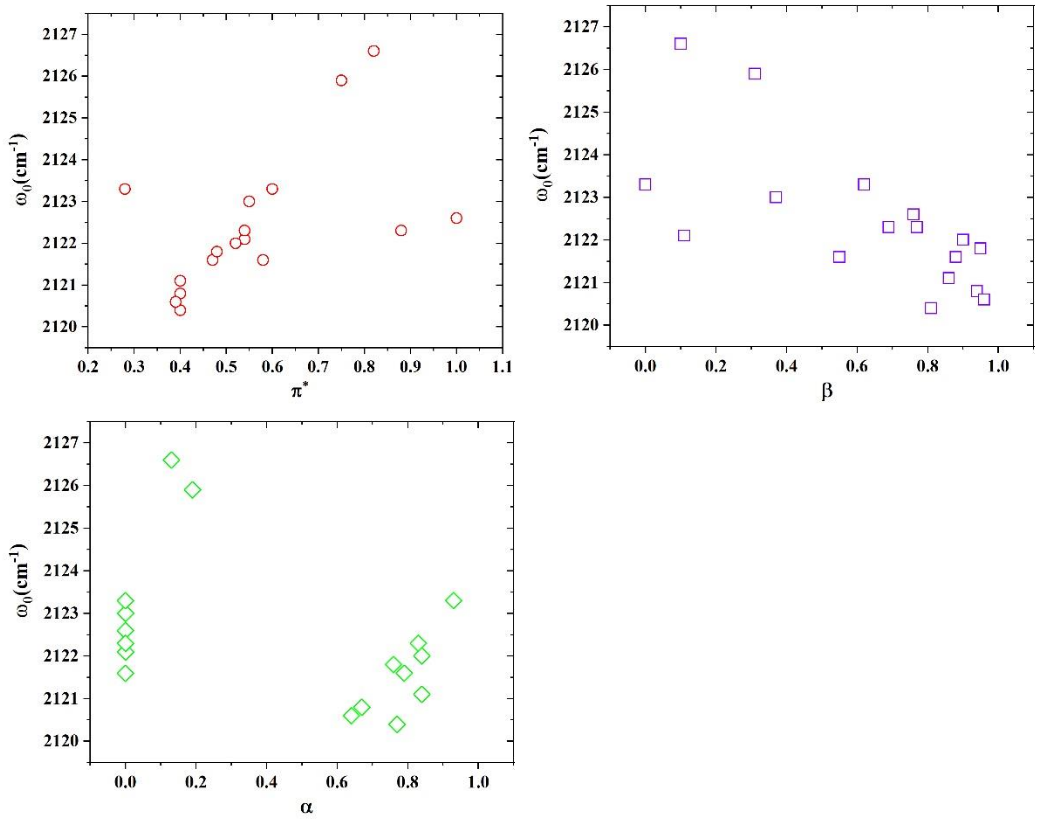


**Figure S4** The NC stretching frequencies of NM5ICI *versus* the density of hydrogen-bond donor groups (mol/mL) in solvents ($R^2$=0.99).

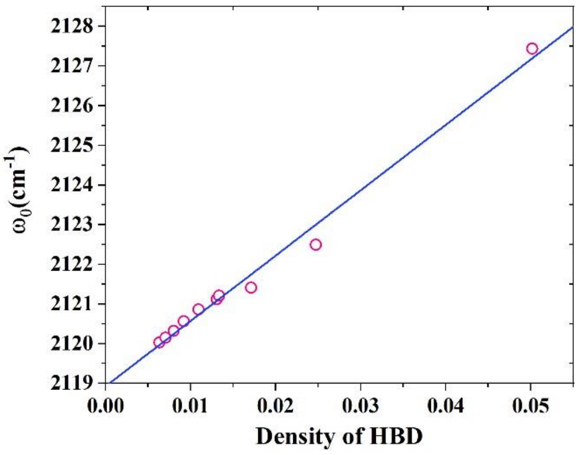

**Figure S5** The NC stretching frequencies of H-bonded NC group *versus* Kamlet-Taft empirical parameter π*, β, or α. Only frequencies obtained in protic solvents (α ≠ 0) were used.

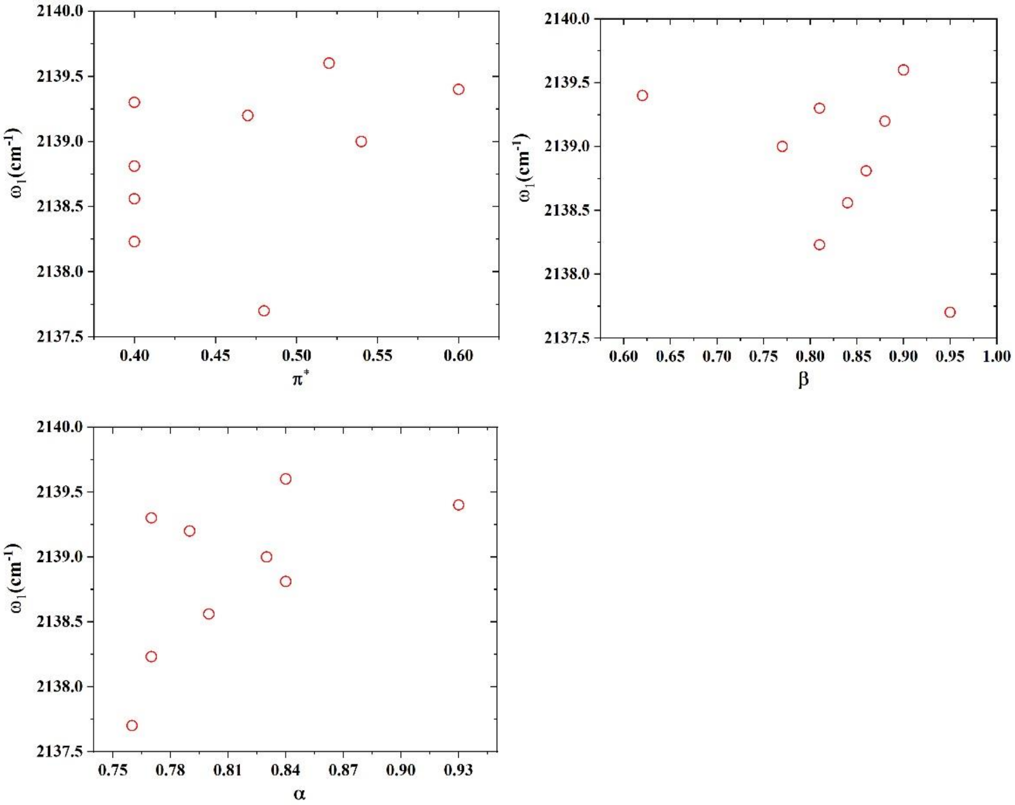


**Figure S6** The NC stretching frequencies of H-bonded NC group *versus* the density of hydrogen bond donors of solvents.

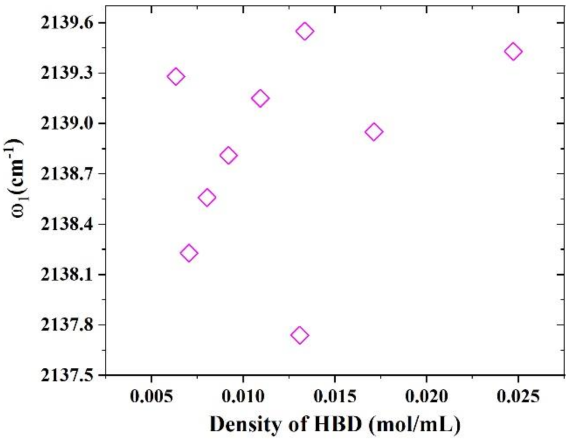

**Figure S7.** The NC stretching vibration of NM5ICI in selected solvents.

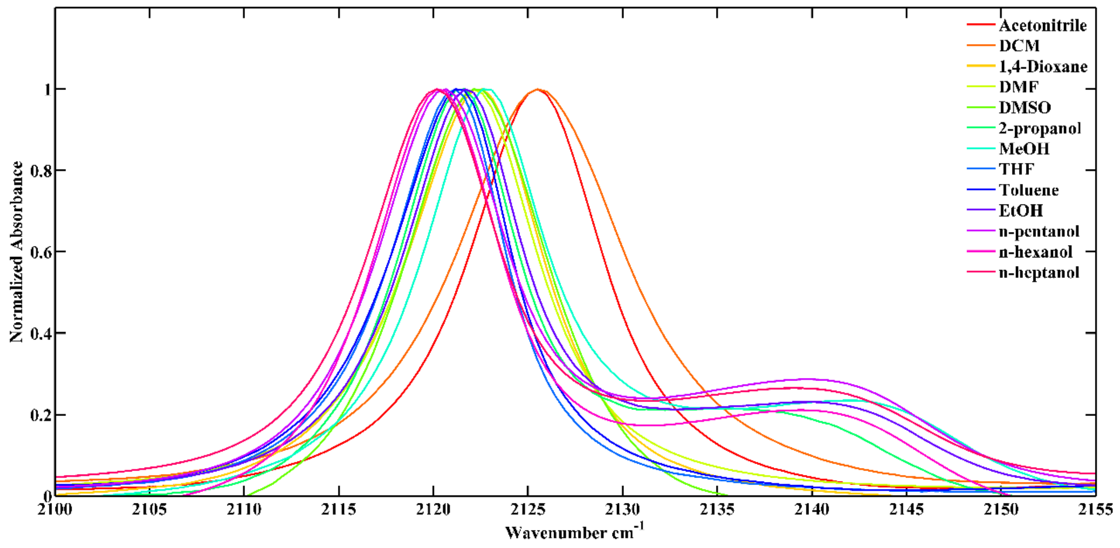

Supplement: Supplementary file 1 [file molecules-24-01379-s001.pdf]
